# Urea Memory: Transient Cell Exposure to Urea Causes Persistent Mitochondrial ROS Production and Endothelial Dysfunction

**DOI:** 10.3390/toxins10100410

**Published:** 2018-10-11

**Authors:** Maria d’Apolito, Anna Laura Colia, Enrica Manca, Massimo Pettoello-Mantovani, Michele Sacco, Angela Bruna Maffione, Michael Brownlee, Ida Giardino

**Affiliations:** 1 Pediatric Research Center, Department of Medical and Surgical Sciences, University of Foggia, 71122 Foggia, Italy; maria.dapolito@unifg.it (M.A.); massimo.pettoellomantovani@unifg.it (M.P.-M.); 2 Department of Clinical and Experimental Medicine, University of Foggia, 71122 Foggia, Italy; annalaura.colia@gmail.com (A.L.C.); angelabruna.maffione@unifg.it (A.B.M.); 3 Pediatric Residency Program, University of Foggia, 71122 Foggia, Italy; enricamanca@hotmail.com; 4 Departement of Pediatrics, Scientific Institute “Casa Sollievo della Sofferenza”, University of Foggia, 71122 Foggia, Italy; m.sacco@operapadrepio.it; 5 Diabetes Research Center, Albert Einstein College of Medicine, New York, NY 10461, USA; Michael.Brownlee@einstein.yu.edu

**Keywords:** urea, uremic memory, CRF, chronic renal failure, ROS, reactive oxygen species, ESRD, end stage renal disease, CVD, cardiovascular disease

## Abstract

Urea at post-dialysis levels induces increased ROS in a number of cell types. The aim of this study was to determine whether urea-induced production of ROS remains elevated after urea is no longer present, and, if it does, to characterize its origin and effects. Human arterial endothelial cells were incubated with 20 mM urea for two days, and then cells were incubated for an additional two days in medium alone. Maximal ROS levels induced by initial urea continued at the same level despite urea being absent. These effects were prevented by either MnSOD expression or by Nox1/4 inhibition with GKT13781. Sustained urea-induced ROS caused a persistent reduction in mtDNA copy number and electron transport chain transcripts, a reduction in transcription of mitochondrial fusion proteins, an increase in mitochondrial fission proteins, and persistent expression of endothelial inflammatory markers. The SOD-catalase mimetic MnTBAP reversed each of these. These results suggest that persistent increases in ROS after cells are no long exposed to urea may play a major role in continued kidney damage and functional decline despite reduction of urea levels after dialysis.

## 1. Introduction

Cardiovascular disease (CVD) is the major cause of death in patients with impaired kidney function [1]. Recent evidence suggests that the process of cardiovascular damage starts very early in the course of progressive chronic kidney disease (CKD), long before end-stage renal disease (ESRD) develops (National Kidney Foundation stages 1–2). Uremic toxins independently contribute to the pathogenesis of CVD [2], in the absence of traditional risk factors such as diabetes mellitus and hypertension. The assumption underlying clinical treatment of ESRD patients is that lowering the level of uremic toxins will reduce the risk of CVD and death [3]. However, current therapeutic modalities used to treat ESRD patients have failed to improve cardiovascular outcomes. Dialysis patients still have a 20–40-fold increased incidence of cardiovascular disease compared with the healthy population [4,5]. The failure of intermittent dialysis to decrease the incidence of CVD suggests that the deleterious effects of pre-treatment uremic toxin levels may persist for days after dialysis has lowered uremic toxin concentration. This effect has been conceptualized as “uremic memory”, a phenomenon analogous to the one described in diabetes as metabolic or hyperglycemic memory [6]. In diabetes, hyperglycemic memory is a concept describing progressive vascular damage years after normalization of hyperglycemia. An analogous short-term memory lasting for days after brief spikes in glycemia also occurs [7,8]. Even transient exposure to pathological concentrations of glucose cause persistent increases in pro-atherogenic gene expression during subsequent periods of normal glycemia, by inducing epigenetic modifications [9]. A key factor in the genesis of metabolic memory is a persistent increase in intracellular ROS production induced by transient hyperglycemia, lasting for days after glucose concentration in normalized [10].

Recently, we showed that urea at concentrations seen in the serum of CRF patients induces endothelial dysfunction, endothelial progenitor cells senescence and insulin resistance. All of these are considered risk factors for cardiovascular disease [11,12,13]. Similar to hyperglycemia, urea can cause all of these pro-atherosclerotic effects by increasing intracellular ROS production in target tissues. We hypothesized that transient exposure to concentrations of urea associated with chronic renal failure may cause a persistent increase in mitochondrial ROS production in arterial endothelial cells during subsequent days of normal uremia. This continuous persistent mitochondrial ROS overproduction after dialysis would cause a progressive accumulation of pathologic changes in arterial endothelial cells, promoting the progression of atherosclerotic lesions into unstable plaques. Thus, similar to hyperglycemia, pathological concentrations of urea could induce long lasting effects on arterial cells. Such a phenomenon could explain the failure of clinical treatment to reduce CVD morbidity and mortality in the CRF population.

## 2. Results

### 2.1. Transient Exposure of Endothelial Cells to a Disease-Relevant Concentration of Urea Induces Increased Mitochondrial ROS Production That Persists for Days after Urea Is Removed

**Figure 1:**
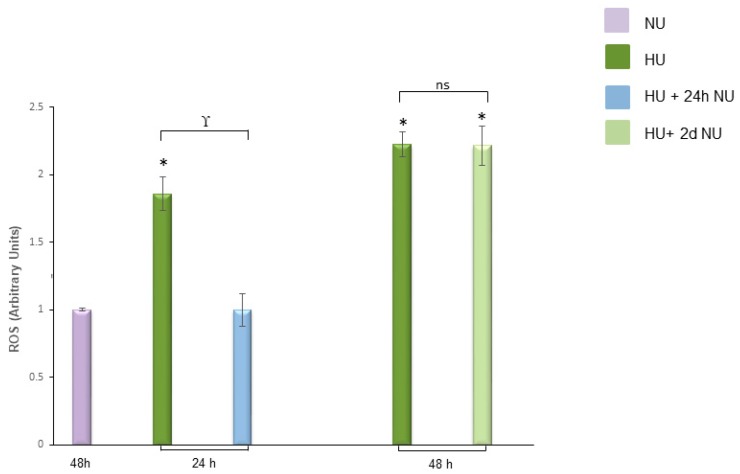
Transient exposure to 20 mM urea induces persistent ROS production. ROS levels in HAEC exposed to 20 mM Urea (HU) for 24 h, 24 h followed by 24 h of subsequent incubation in media without urea, 48 h, and 48 h followed by two days of subsequent incubation in media without urea (HU+ 2dNU). ROS levels were measured by CM-H2DCFDA. Data are the mean ± S.E. from five independent experiments. * *p* < 0.05 compared to cells not treated with urea, γ *p* < 0.05 HU compared with HU+ 24 h NU. ns: not significant for cells treated with HU 48 h compared to cells treated 48 h with HU + two days of NU.

To investigate whether transient exposure to pathological concentrations of urea can induce a persistent increase of ROS concentration, human aortic endothelial cells (HAEC) were exposed to 20 mM Urea (HU) up to 48 h. The cells were then maintained in media without urea (NU) for two more days. Twenty millimolar urea is the minimal urea concentration that induces maximum ROS production in endothelial cells [12]. In the cells incubated with HU for 24 h, the intracellular ROS concentration increased by 2.1-fold (Figure 1, bar 2) and then returned to basal levels by 24 h after the urea was removed (Figure 1, bar 3). Mannitol, used as an osmotic control, had no effect on the intracellular ROS production (Figure S1). Similarly, in endothelial cells incubated with HU for 48 h, ROS were increased 2.2-fold (Figure 1, bar 4), but persisted without significant change for two days after the urea was removed (Figure 1, bar 5). Although 24 h of exposure to HU was sufficient to cause an increase in ROS concentration, 48 h of incubation with HU were necessary to induce an increase of ROS production that persisted for two subsequent days in the absence of urea. Since ROS have a very short half-life, their increased concentration after two days without urea suggests that transient exposure to HU activates self-maintaining ROS generating mechanisms that maintain increased intracellular ROS production even when the initial pro-oxidative stimulus is removed.

### 2.2. Cytosolic and Mitochondrial ROS Generating Mechanisms Initiate the Persistent ROS Production Induced by Transient Exposure to 20 mM Urea in Endothelial Cells

**Figure 2:**
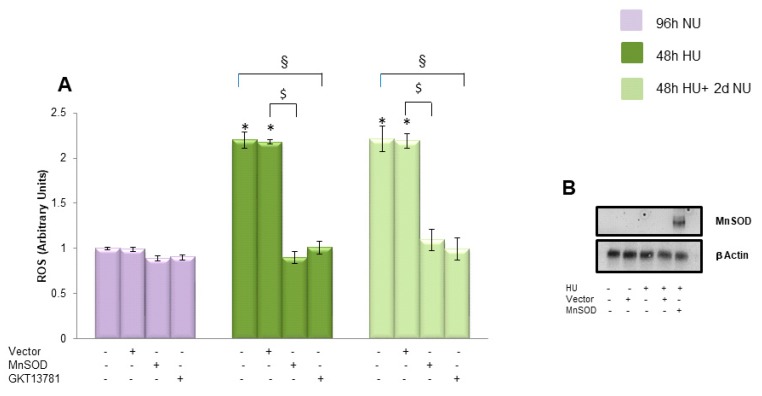
Cytosolic and mitochondrial ROS generating mechanisms start the persistent ROS production induced by transient exposure to 20 mM urea in endothelial cells. (**A**) In the indicated groups, HAEC were infected with MnSOD, or control adenoviral vectors before transient exposure to 20 mM glucose, or incubated for 48 h in 20 mM urea in the presence of the NADPH oxidase inhibitor GKT137831. ROS levels were measured by CM-H2DCFDA. (**B**) MnSOD protein level detected in HAEC 96 h after cells were infected with MnSOD adenovirus (MOI 500) compared to MnSOD level detected in cells infected with empty vector. Data are the mean ± S.E. from five independent experiments. * *p* < 0.05 compared to cells treated in the same conditions but not exposed to urea, $ *p* < 0.05 cells transfected with the empty vector compared to cells over expressing MnSOD, § *p* < 0.05 cells not transfected or not treated with the inhibitor compared to cells treated with GKT13781.

Since 20 mM urea increases ROS production in HAECs through the activation of both mitochondrial and cytosolic ROS generating mechanisms [11,12], we investigated how the inhibition of either affected the persistent ROS generation induced by transient HU. To evaluate the role played by mitochondria in the generation of persistent ROS production induced by urea, we overexpressed manganese superoxide dismutase (MnSOD), the mitochondrial isoform of this enzyme [14]. As shown in Figure 2, bar 11, overexpression of MnSOD prevented the persistent ROS production induced by prior exposure to HU. MnSOD overexpression was confirmed by Western blot (Figure 2B). Next, we explored the involvement of cytosolic mechanisms in the activation of the persistent ROS production induced by transient urea exposure. Since NADPH oxidases are major cytosolic sources of superoxide in vascular cells [15], and urea increased NADPH oxidase activity in endothelial cells, we treated HAEC with the most specific NADPH Oxidase 1/4 (Nox1/4) inhibitor currently available, GKT137831 [12]. As shown in Figure 2, bar 12, GKT137831 also prevented the persistent ROS production induced by transient HU. Together, these findings demonstrate that both mitochondrial and cytosolic ROS generating mechanisms are involved in initiating the persistent ROS over-production induced by transient exposure to 20 mM Urea.

**Figure 3:**
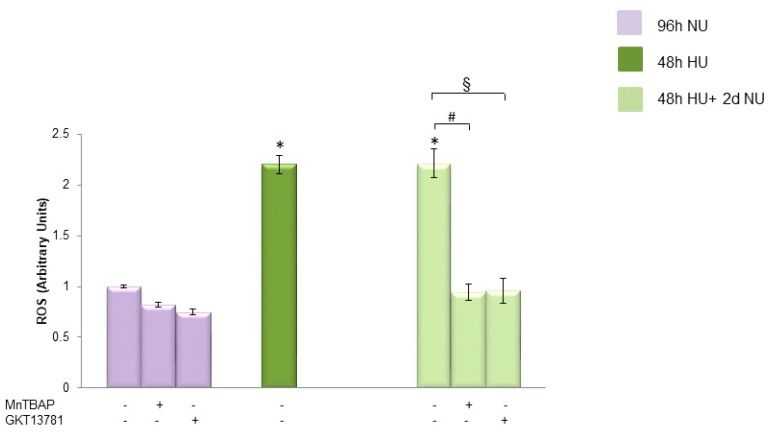
The persistent ROS production induced by transient exposure to urea was reversed by both MnTBAP and GKT13781. ROS levels in HAEC exposed to 20 mM Urea (HU) for 48 h 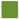, and to 20 mM urea for 48 h followed by two days of subsequent incubation in media without urea (NU) 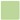 ± MnTBAP or GKT13781. ROS levels were measured by CM-H2DCFDA. Data are the mean ± S.E. from five independent experiments. * *p* < 0.05 compared to cells treated in the same conditions but not exposed to urea, # *p* < 0.05 cells not treated with the inhibitors compared to cells treated with MnTBAP, § *p* < 0.05 cells not treated with the inhibitors compared to cells treated with GKT13781.

Next, to investigate the source of the urea induced persistent ROS production in the absence of urea, we treated the cells with either the MnSOD mimetic MnTBAP or GKT137831 for two days after the urea was removed. As shown in Figure 3, both MnTBAP and GKT137831 were able to reverse the urea-induced persistent ROS production, indicating that both mitochondrial and NOX1/4 are responsible for maintaining the increased ROS production in the cells after the urea is removed.

### 2.3. Urea-Induced Mitochondrial Dysfunction Maintains Persistent Mitochondrial ROS Production in Endothelial Cells after Transient Exposure to High Urea Concentrations 

#### 2.3.1. Transient Exposure to 20 mM Urea Induces a Long-Lasting Reduction of mtDNA Copy Number in Endothelial Cells

We next investigated the mechanism by which transient incubation with HU can cause persistent ROS production in endothelial cells after the initial urea pro-oxidative stimulus has been removed. Since mtDNA damage causes increased ROS production [16,17], we investigated the effect of urea on mtDNA copy number in endothelial cells

**Figure 4:**
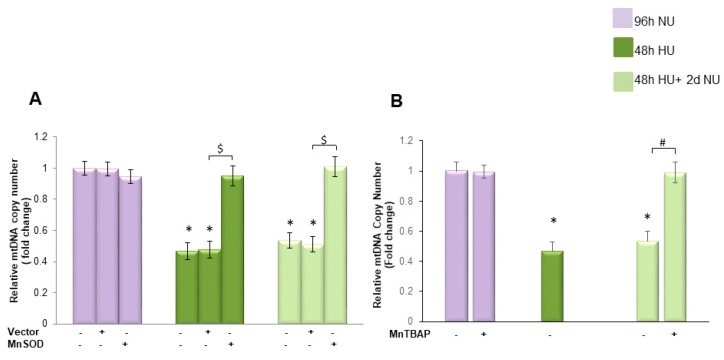
Transient exposure to 20 mM urea induces a persistent reduction of mtDNA copy number in endothelial cells that is reversed by MnTBAP treatment. (**A**) HAEC were exposed to 20 mM Urea (HU) for 48 h 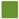, and to 20 mM urea followed by two days of subsequent incubation in media without urea (NU) 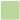. In the indicated groups, cells were infected with MnSOD before transient exposure to urea (A), or were treated with MnTBAP during the two days of subsequent incubation in media alone (**B**). mtDNA copy number was measured by a quantitative Real-time polymerase chain reaction (PCR)-based method. Data are the mean ± S.E. from seven independent experiments. * *p* < 0.05 compared to cells treated in the same conditions but not exposed to urea, $ *p* < 0.05 cells transfected with the empty vector compared to cells over expressing MnSOD, # *p* < 0.05 cells not treated with the inhibitor compared to cells treated with MnTBAP.

As shown in Figure 4A, bar 4, in cells exposed to 20 mM urea for 48 h, the mean mtDNA copy number was decreased by 55% compared to the mean mtDNA copy number found in the control cells. This reduction in mtDNA copy number persisted unchanged for two days in the absence of urea, indicating that transient HU can induce long lasting reduction in mtDNA copy number (Figure 4, bar 7). Overexpression of MnSOD completely prevented the persistent reduction of mtDNA copy number induced by transient cell exposure to 20 mM urea, indicating that the persistent reduction in mtDNA is maintained by continued overproduction of ROS caused by the initial urea-induced mitochondrial ROS overproduction in endothelial cells.

To investigate the role played by the persistent ROS production in the long-lasting mtDNA loss in the absence of urea, we treated the endothelial cells with MnTBAP for two days after the urea was removed. As shown in Figure 4B, MnTBAP completely reversed the long lasting effect of urea on mtDNA copy number, indicating that mtDNA is reduced in the absence of urea by the persistent ROS over-production.

#### 2.3.2. Transient Exposure to 20 mM Urea Causes a Long-Lasting Reduction of Electron Transport Chain Component Expression

**Figure 5:**
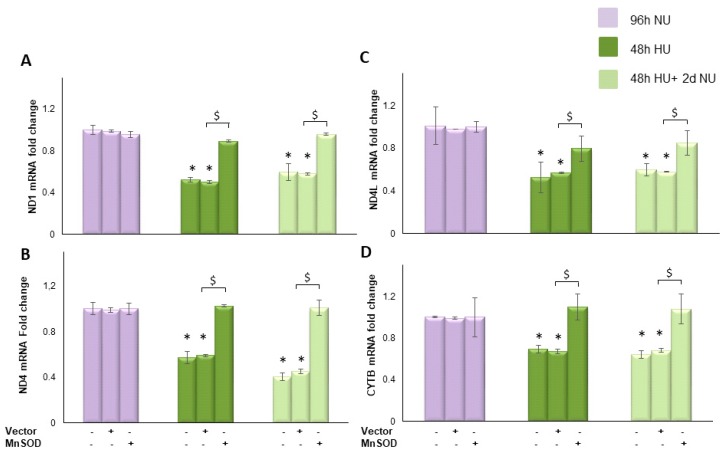
Transient exposure to 20 mM urea causes a persistent reduction in mtDNA transcription, which is normalized by MnSOD. HAEC with or without MnSOD infection were exposed to 20 mM urea for 48 h 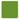, or to 20 mM urea for 48 h followed by two days of subsequent incubation in media alone 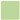. The mRNA expression was measured by Real time PCR: (**A**) ND1; (**B**) ND4; (**C**) ND4L; and (**D**) cytochrome b. Data are the mean ± S.E. from five independent experiments. * *p* < 0.05 compared to cells treated in the same conditions but not exposed to urea, $ *p* < 0.05 cells transfected with the empty vector compared to cells over expressing MnSOD.

We next evaluated the effect of transient HU on the expression of mitochondrial genes ND1, ND4, ND4L and cytochrome b to determine the effects of the urea-induced reduction of mtDNA copy number on mitochondrial function. In the cells incubated with HU for 48 h, the expression of ND1, ND, ND4L and cytochrome b were reduced by 47.5%, 42.3%, 47.8%, and 31%, respectively (Figure 5A–D, bar 4) compared to control. The urea-induced reduction in the expression of each one of these mitochondrial genes lasted unchanged for two days after the urea was removed (Figure 5A–D, bar 7), indicating that transient HU can persistently compromise the electron transport system. This long lasting effect of urea on expression of proteins encoded in the mitochondrial genome was prevented by the overexpression of MnSOD (Figure 5A–D, bar 9). These results suggest that transient exposure to urea caused a self-sustaining ROS generating system that continued to overproduce ROS in the absence of urea.

**Figure 6:**
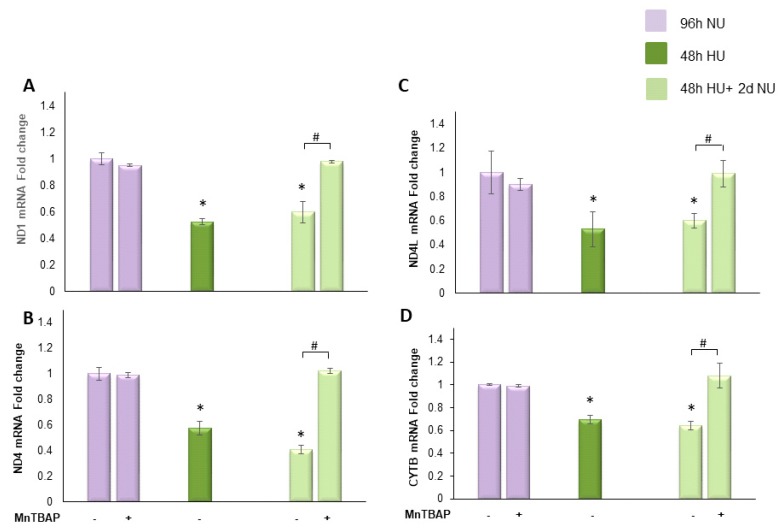
MnTBAP reverses the long lasting reduction of mitochondrial electron transport chain protein transcription caused by transient exposure to urea. HAEC were exposed to 20 mM urea 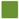 for 48 h, or to 20 mM urea for 48 h followed by two days of subsequent incubation in media alone, 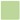, with or without MnTBAP. The mRNA expression was measured by Real time PCR: (**A**) ND1; (**B**) ND4; (**C**) ND4L; and (**D**) cytochrome b. Data are the mean ± S.E. from five independent experiments. * *p* < 0.05 compared to cells treated in the same conditions but not exposed to urea, # *p* < 0.05 cells not treated with the inhibitor compared to cells treated with MnTBAP.

To understand the role played by the urea-induced persistent ROS production in maintaining reduced expression of the indicated mitochondrial DNA encoded proteins in the absence of urea, we treated the cells with MnTBAP for two days following urea incubation. As shown in Figure 6, MnTBAP reversed each of the urea memory effects on the transcription of the mitochondrial proteins, demonstrating that these persistent post-urea effects are maintained by persistent ROS production following removal of urea.

#### 2.3.3. Transient Exposure to 20 mM Urea Causes a Long-Lasting Dysregulation of Mitochondrial Dynamics in Human Aortic Endothelial Cells

**Figure 7:**
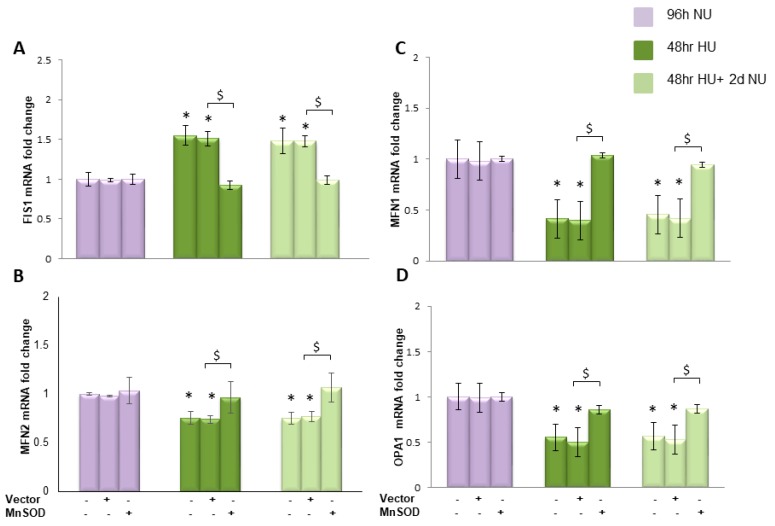
Transient exposure to 20 mM urea causes a persistent reduction in transcription of mitochondrial fusion and increase in fission proteins that lasts two days in the absence of urea. mRNA expression was evaluated by Real time PCR: (**A**) Fis1; (**B**) Mfn1; (**C**) Mfn2; and (**D**) OPA1. Data are the mean ± S.E. from five independent experiments. * *p* < 0.05 compared to cells treated in the same conditions but not exposed to urea, $ *p* < 0.05 cells transfected with the empty vector compared to cells over expressing MnSOD.

Increased mitochondrial fission induces increased ROS production [18]. Therefore, we investigated whether transient HU alters the mitochondrial fusion–fission equilibrium. The effect of 20 mM urea on the mRNA expression of fission protein 1 (Fis1), Mitofusins (Mfn1 and Mfn2), and the dynamin family GTPase optic atropy 1 (OPA1) were evaluated. Fis1 is responsible for mitochondrial fission, while mitofusins and OPA1 regulate mitochondrial fusion [19]. As shown in Figure 7A, bar 4, 48 h exposure to HU increased the expression of Fis1 by 1.5-fold and this increased expression lasted unchanged for two days after the urea was removed (Figure 7A, bar 7). At the same time, transient exposure to HU reduced the expression of Mif1, Mif2 and OPA1 by 0.41-, 0.66- and 0.55-fold, respectively (Figure 7B–D, bar 4). Each one of these urea-induced changes in gene expression persisted for two days in the absence of urea (Figure 7B–D, bar 7). These results demonstrated that transient HU induces a long lasting change in mitochondrial dynamics, increasing mitochondrial fission and reducing mitochondrial fusion. Overexpression of MnSOD completely prevented this persistent reduction in transcription of mitochondrial fusion and increase in fission proteins, suggesting that this was mediated by the initial ROS overproduction induced by HU.

**Figure 8:**
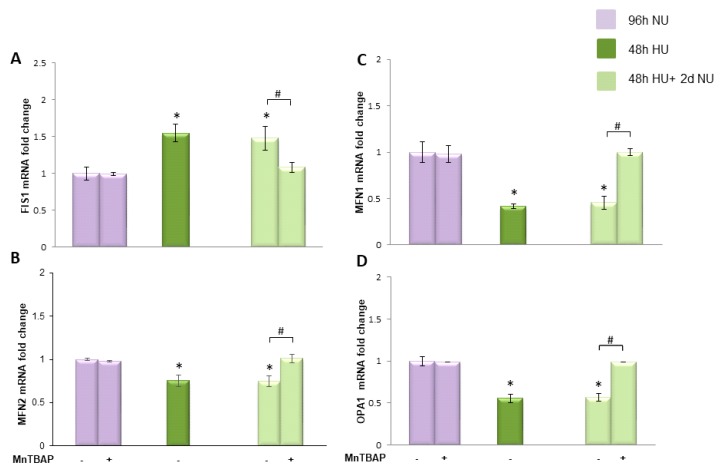
MnTBAP reverses the persistent reduction in transcription of mitochondrial fusion and increase in fission proteins caused by transient exposure to urea. HAEC were exposed to 20 mM urea for 48 h 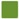 or to 20 mM urea for 48 h followed by two days of subsequent incubation in media alone, with or without MnTBAP 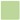. The mRNA expression was measured by Real time PCR: (**A**) Fis1; (**B**) Mfn1; (**C**) Mfn2; and (**D**) OPA1. Data are the mean ± S.E. from five independent experiments. * *p* < 0.05 compared to cells treated in the same conditions but not exposed to urea, # *p* < 0.05 cells not treated with the inhibitor compared to cells treated with MnTBAP.

To investigate the mechanism responsible for maintaining the persistent dysregulation in transcription of mitochondrial dynamic related proteins when the urea is removed, we normalized ROS during the post-urea persistent ROS period and compared the results with those where ROS were normalized during urea exposure. We treated HAEC with MnTBAP for two days following urea treatment and measured the mRNA expression. Figure 8 shows that MnTBAP was able to reverse completely the long lasting effects of urea on mitochondrial dynamic protein transcription, indicating that persistent ROS production was responsible for maintaining these effects in the absence of urea.

#### 2.3.4. Transient HU Causes Endothelial Cell Dysfunction Lasting for 2 Days Despite Urea Normalization

**Figure 9:**
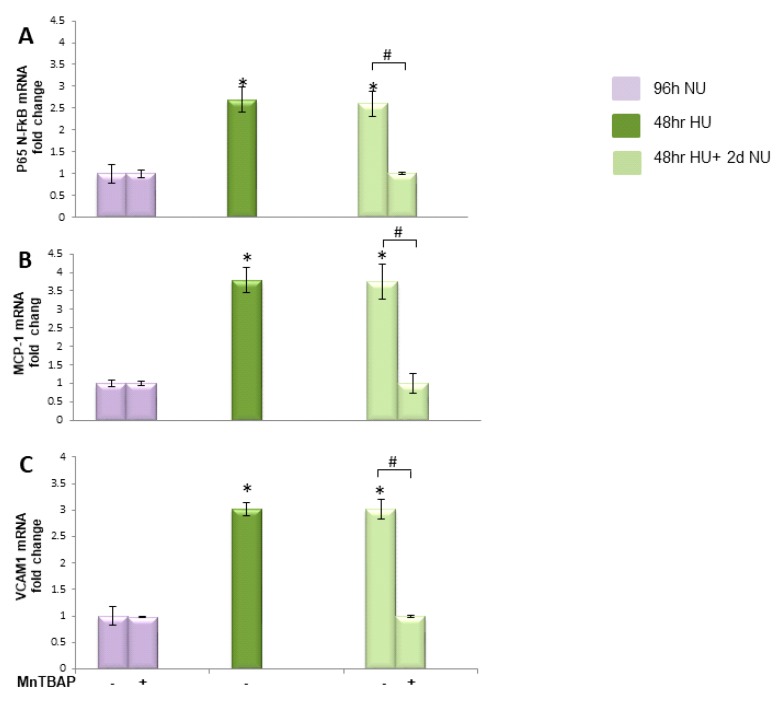
Transient exposure to 20 mM urea induces persistent expression of inflammatory markers and MnTBAP reverses these. mRNA levels of NF-κB p65 subunit (**A**); VCAM1 (**B**); and MCP-1 (**C**) in HAEC exposed to 20 mM urea for 48 h 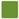 or to 20 mM urea for 48 h followed by two days of subsequent incubation in media alone 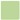 ± MnTBAP. Data are the mean ± S.E. from five independent experiments. * *p* < 0.05 compared to cells treated in the same conditions but not exposed to urea, # *p* < 0.05 cells not treated with the inhibitor compared to cells treated with MnTBAP.

In diabetes, it has been shown that the persistent rise of ROS production induced by transient hyperglycemia in endothelial cells causes a persistent increase in the expression of pro-atherogenic genes during subsequent periods of normal glycaemia [7,10]. To determine whether the persistent ROS production induced by transient HU can cause similar effects in HAEC, the expression of the major inflammatory mediator NFκB subunit p65 was measured in cells exposed to 20 mM urea for 48 h and then maintained for two more days in media without urea. As shown in Figure 9A, bar 4, the 2.8-fold increase in p65 mRNA expression induced by exposure to 20 mM urea for 48 h persisted unchanged for two days in the absence of urea. Similarly, transient HU induced a persistent increase in the expression of the NFκB-specific inflammatory target genes MCP-1 and VCAM1 [9,20]. The 3.6- and 3.0-fold increases in MCP-1 and VCAM1, respectively, remained increased for two days after the urea was removed (Figure 9B,C, bars 4). These data indicate that urea induced a persistent activation of NFκB. To test the hypothesis that the persistence of these changes in pro-atherogenic genes expression are due to persistently increased mitochondrial ROS production caused by urea-induced mitochondrial dysfunction, cells exposed to HU for 48 h were treated with the MnSOD mimetic MnTBAP for two days after the urea was removed. MnTBAP completely reversed the urea-induced changes in NFκBp65, MCP-1, and VCAM1 gene expression (Figure 9A–C, bars 5). These results demonstrate that persisting changes in endothelial cell pro-atherogenic gene expression are mediated by ROS over production that continued in the absence of urea.

## 3. Discussion

The persistent adverse effects of prior sustained exposure to hyperglycemia persisting for years after hyperglycemia has been ameliorated is defined as “metabolic memory” [7] Accumulating evidence supports the concept that even prior exposure to hyperglycemia for hours causes persistent ROS lasting for days of subsequent normal glucose, triggering vascular dysfunction and damage [21,22] by inducing epigenetic modifications [10,23].

Similar to glucose, urea at disease relevant concentrations has a direct vascular toxicity [24,25], inducing endothelial dysfunction by increasing intracellular ROS production through the activation of both mitochondrial and cytosolic ROS generating mechanisms [11,12]. In the present report, we have shown that transient exposure to 20 mM urea induces ROS production in human aortic endothelial cells that persists for days after urea is removed. Thus, a cellular memory for urea-induced oxidative stress exists in endothelial cells. Inhibition of either mitochondrial or cytosolic ROS generating mechanisms prevents completely this urea memory effect, indicating that both are activated by transient exposure to urea and both initiate this long lasting ROS over-production. These data are consistent with the recent finding that NADPH oxidase 4 (NOX4), although considered a direct source of increased ROS, appears to act indirectly by increasing mitochondrial ROS production. NOX4 is constitutively active and associates with mitochondria, inhibiting mitochondrial biogenesis and function [26]. Knockout of NOX4 increases mitochondrial biogenesis and maximal respiratory capacity dramatically, which would prevent substrate driven increased mitochondrial ROS production [26]. Thus, inhibition of NOX4 most likely completely reduced urea-induced increased ROS indirectly, while inhibition of mitochondrial ROS with MnSOD directly reduced increased ROS. 

This initial increase of ROS induced by transient exposure to urea affected mitochondrial function and dynamics, resulting in continued ROS overproduction in endothelial cells that persisted for two days after urea is removed.

**Figure 10:**
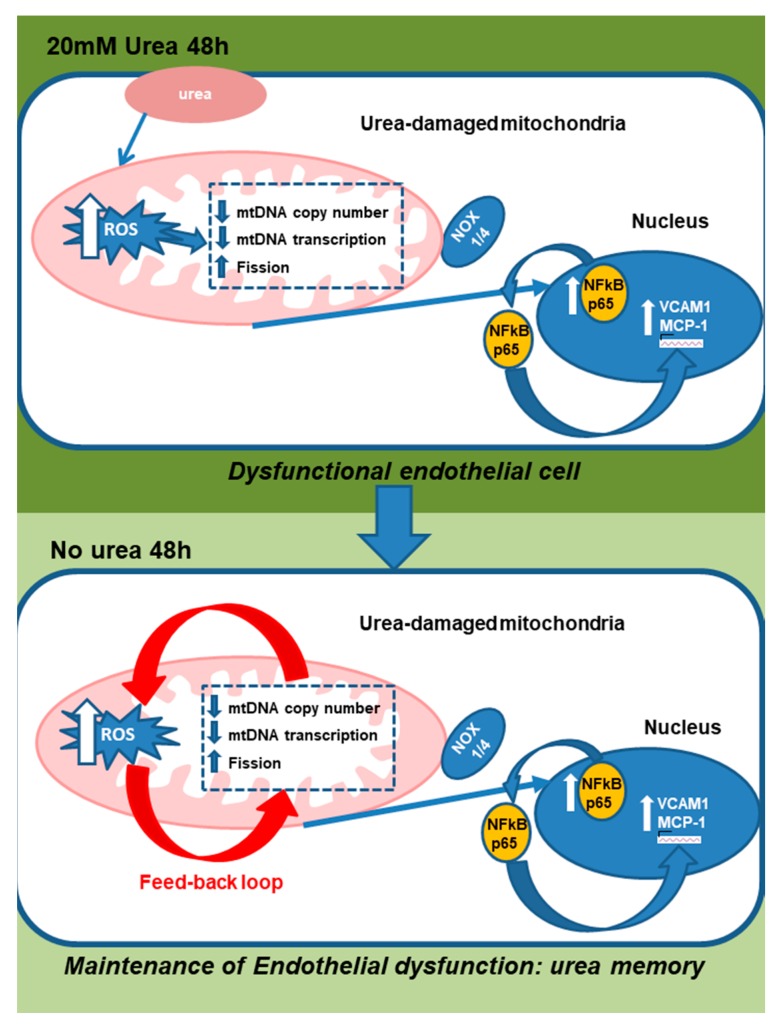
Scheme showing that urea-induced mitochondrial damage initiates a vicious cycle of ROS production that continues to self-propagate after the urea insult is terminated.

In the HAEC exposed transiently to 20 mM urea, the mtDNA copy number and the transcripts of mtDNA-encoded proteins were significantly reduced. The human mitochondrial genome is a 16.6 kb circular double-stranded DNA coding for proteins essential for cellular respiration and normal mitochondrial function [27]. Reduced mtDNA copy number has been shown to attenuate the transcripts of mtDNA-encoded proteins, impairing the integrity and the function of the electron transport chain system. This causes persistent ROS production [27,28,29]. In the present study, we have shown that transient urea exposure significantly reduces transcription of the ND1, ND4L and ND4 subunits of Complex I, which has been recently recognized to be the mitochondrial source of deleterious ROS [30]. Complex I derived ROS can readily react with mitochondrial DNA or other matrix components vulnerable to oxidative damage. In contrast, Complex III-derived ROS serve as second messengers in cellular signaling [30]. In addition, the present study shows that transient exposure of cells to HU also affects the transcription of proteins regulating mitochondrial fission and fusion, resulting in a persistent shift from fusion to fission. Accumulating evidence suggests that prolonged disruption of mitochondrial dynamics with persistent mitochondrial fragmentation is caused by oxidative stress, and is responsible for persistent excessive ROS production and endothelial dysfunction [18,31,32]. In the present study, persistence of reduced mtDNA copy number, electron transport chain transcripts and mitochondrial fission proteins were all reversed by treating post-urea cells with MnTBAP. Together, these results show that the urea-induced mitochondrial damage initiates a vicious cycle of ROS production that continues to self-propagate even after the urea insult is terminated. This persistent ROS production causes an increased expression of inflammatory markers in endothelial cells that also last for two days after the urea is removed (Figure 10). Although our study proves that dysfunctional mitochondria are the source of the increased ROS production that persists when the urea is removed, our data do not exclude the possibility that a cytosolic source of ROS could also be persistently activated by the transient exposure to urea, and thus also contribute to the self-propagating cycle of ROS production observed in absence of the initial urea stimulus. Our study also did not investigate how long the urea memory effect can last, and what mechanisms are involved in its termination.

In CKD patients, recent studies reported that the mtDNA copy number was significantly associated with clinical outcome in dialysis patients [33,34]. A low mtDNA copy number, in peripheral blood mononuclear cells, correlates with increased oxidative stress and higher mortality rates in patients undergoing dialysis, and mtDNA copy number is already decreased in patients with stage 3–4 CKD. The observation that the uremic toxin hippurate increased mitochondrial fission in endothelial cells [35] suggests that additive or synergistic effects of urea with other uremic toxins may induce uremic memory. However, in the present study, urea alone was sufficient to cause uremic memory.

In our in vitro system, cell exposure to 20 mM urea shorter than 48 h did not establish uremic memory. Consistent with this observation, the survival in patients on daily hemodialysis has been documented to be 2-fold greater than in patients dialyzed less frequently [36]. We speculate that daily hemodialysis removes urea and other uremic toxins before they can cause mitochondrial damage and initiate self-propagating ROS-generating systems.

The findings described in the present study provide a conceptual framework for further research into the underlying mechanisms that contribute to the enhanced cardiovascular risk associated with chronic renal failure.

## 4. Materials and Methods

### 4.1. Cell Culture Conditions

Confluent primary human aortic endothelial cells (HAECs) from Cambrex, (East Rutherford, NJ, USA) (passages 2–5) were maintained in EBM-2 medium (from Lonza, San Diego, CA, USA) with 0.4% fetal bovine serum. Cells were incubated with either 20 mM urea or with 20 mM mannitol used as osmotic control, for 24 or 48 h. Then, the urea was removed and the cells were maintained in culture for two more days. The urea used in these experiments was certified to be free of LPS and heavy metals (Sigma Aldrich, St. Louis, MO, USA). In a group of experiments, cells were infected with MnSOD adenovirus or control adenovirus at an MOI (multiplicity of infection) of 500, 4 h before addition of 20 mM urea containing medium. In a different group, after the urea was removed, cells were maintained for two more days in basal medium in the presence or absence of 200 μM MnTBAP (Mn(III)tetrakis(4-benzoic acid)) porphyrin chloride, a cell permeable mimetic of manganese superoxide dismutase (Calbiochem-Merck KGaA, Darmstadt, Germany) [13,37].

### 4.2. Adenoviral Vectors

SOD2 was cloned into the shuttle vector pAd5CMVK-NpA, and both adenoviral vectors and empty control virus were prepared by the Gene Transfer Vector Core at the University of Iowa, as described previously [11]. HAEC were infected with MnSOD, or control adenovirus at an MOI (multiplicity of infection) of 500, 4 h before addition of either 20 mM urea containing medium or control medium [11].

### 4.3. NADPH Oxidase Inhibition

Cells were cultured for the time indicated in the presence or absence of 20 mM urea with or without the highly specific NADPH oxidase inhibitor GKT137831 (Cayman Chemical, Ann Arbor, MI, USA) [12] at concentrations of 10 mM. GKT137831 was dissolved in 0.1% ethanol and was added to the cells 20 min before urea addition. In the indicated experiments, GKT137831 was added to the cells after the urea was removed

### 4.4. Measurement of ROS Generation

Treated cells seeded in a 96-well plate were incubated with 10 μmol/L CM-H2DCFDA (Molecular Probes-Life Technology, Brooklyn, NY, USA) for 45 min at 37 °C, and the intracellular formation of ROS was measured at excitation/emission wavelengths of 485/530 nm using a Wallac 1420 Fluorescent Plate Reader.

### 4.5. Determination of mtDNA Content by Real-Time Quantitative Polymerase Chain Reaction

QIAamp DNA Mini kits (Qiagen, Valencia, CA, USA) was used to extract genomic DNA from HAEC cell culture. A quantitative real-time polymerase chain reaction (PCR)-based method was employed to measure relative mtDNA copy number by. In brief MT-ND1 and CYTB gene in mtDNA were amplified by using two primer pairs was used for the amplification of the. Another primer pair was used for the amplification of the single-copy nuclear gene human globulin (HGB). All samples were assayed in duplicate on optical 96-well reaction plates on a CFX96 Touch Real-Time PCR detection System using iQ SYBR green supermix (Bio-Rad Laboratories Inc., Hercules, CA, USA). CFX manager software were used to perform experiment setup and data analysis. To avoid possible position effects, the PCRs for mtDNA and HGB were performed every time on separate 96-well plates with the same samples in the same well positions. In each run, a standard curve of a diluted reference DNA was included. For each standard curve, one reference DNA sample was serially diluted 1:2 to produce a seven-point standard curve between 0.3125 and 20 ng of DNA. For each sample, we determined the ratio of mtDNA copy number to HGB copy number using the standard curves.

### 4.6. RT Reaction and Real-Time Quantitative PCR

The RNeasy Mini Kit (QIAGEN Milan, IT was used to extract the total RNA from treated cells), following the manufacturer’s instructions. One percent agarose gel electrophoresis was used to assay the purity of mRNA. mRNA was quantified with a Nanodrop ND-2000 spectrophotometer (Nanodrop Technologies, Wilmington, DE, USA). The mRNA reverse transcription was performed by SuperScript IV First Strand Synthesis System (Life Technology, Brooklyn, NY, USA. CFX96 Touch™ Real-Time PCR Detection System with iQ SYBR green supermix (Bio-Rad, Hercules, CA, USA) was used. The relative amount of the transcripts of four mt genes (ND1, ND4, ND4L and CYB) and expression levels of p65 NFκB, VCAM1, and MCP-1 mRNA were evaluated. The expression level of two endogenous reference genes, glyceraldehyde-3-phosphate dehydrogenase (GAPDH) and β-actin (ATCB) were analyze to normalize for nonspecific variations in real-time PCR, PCR conditions were as follows: 7 min at 95 °C and 45 cycles of 30 s at 95 °C and 30 s at 60 °C. CFX manager software (Bio-Rad, Hercules, CA, USA) was used to analyze gene expression.

### 4.7. Statistical Analysis

One-factor ANOVA was used to compare the means of all groups. The Tukey–Kramer multiple-comparisons procedure was used to determine which pairs of means were different. *p* value less than 0.05 was considered statistically significant.

## Supplementary Materials

The following are available online at http://www.mdpi.com/2072-6651/10/10/410/s1, Figure S1: Mannitol did not increase intracellular ROS.

## Nonstandard Abbreviations

CRF	chronic renal failure
ROS	reactive oxygen species
ESRD	end stage renal disease
CVD	cardiovascular disease
HU	High urea; NU No urea
