# Supplementary material for: Urea Memory: Transient Cell Exposure to Urea Causes Persistent Mitochondrial ROS Production and Endothelial Dysfunction
Source: Toxins (Basel). 2018 Oct 11;10(10):410. doi: 10.3390/toxins10100410 (PMC6215169; doi:10.3390/toxins10100410)
Supplement: Supplementary file 1 [file toxins-10-00410-s001.pdf]

Supplementary Materials: Urea Memory: Transient Cell Exposure to Urea Causes Persistent Mitochondrial ROS Production and Endothelial Dysfunction

Maria d'Apolito, Anna Laura Colia, Enrica Manca, Massimo Pettoello-Mantovani, Michele Sacco, Angela Bruna Maffione, Michael Brownlee and Ida Giardino

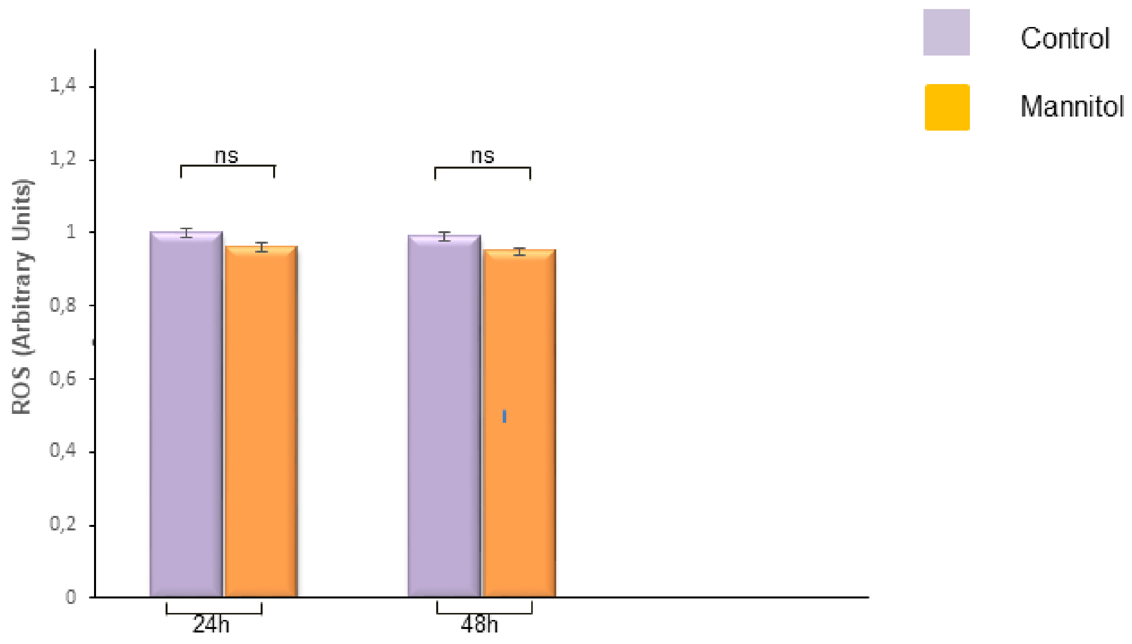

Figure S1. Mannitol did not increase intracellular ROS. ROS levels in HAEC exposed to 20 mM mannitol for 24 h and 48 h. ROS were measured by CM-H2DCFDA. Data are the mean ± S.E. from 5 indepent experiments. ns: not significant cells treated with mannitol comapared to control.
